# Comparison of Cold-Knife Conization versus Loop Electrosurgical Excision for Cervical Adenocarcinoma In Situ (ACIS): A Systematic Review and Meta-Analysis

**DOI:** 10.1371/journal.pone.0170587

**Published:** 2017-01-26

**Authors:** Yanming Jiang, Changxian Chen, Li Li

**Affiliations:** 1 Department of Gynecology, Liuzhou People’s Hospital, Liuzhou, China; 2 Department of Gynecologic Oncology, Affiliated Tumor Hospital of Guangxi Medical University, Nanning, China; Tata Memorial Centre, INDIA

## Abstract

**Objective:**

The objective of this systematic review was to conduct a more comprehensive literature search and meta-analysis of original studies to evaluate the efficacy and safety of the loop electrosurgical excision procedure (LEEP) versus cold-knife conization (CKC) in conservative surgical treatment of cervical adenocarcinoma in situ (ACIS) for women who have not completed childbearing.

**Methods:**

Systematic searches were conducted in the PUBMED, EMBASE, Cochrane, and China National Knowledge Infrastructure (CNKI) databases to identify all potential studies involving patients with ACIS treated with LEEP versus CKC published until December 2015.

**Results:**

Eighteen retrospective studies were included in this systematic review. All the 18 included studies reported the rate of positive margins, and the results of the individual studies varied. The positive margins were 44% (267/607) after LEEP and 29% (274/952) after CKC. The pooled meta-analysis exhibited significantly different outcome (RR, 1.55; 95% CI, 1.34–1.80, P<0.00001) without significant heterogeneity (P = 0.34). The residual rate following LEEP was 9.1% (17/186) and 11% (39/350) after CKC in re-cone or hysterectomy cases. Recurrent ACIS following LEEP was reported in 10 of 142 (7.0%) cases compared to 10 of 177 (5.6%) cases following CKC. There were no significant differences in the residual rate (RR, 1.02; 95% CI, 0.60–1.72, P = 0.95) or recurrence rate (RR, 1.13; 95% CI, 0.46–2.79; P = 0.79) between the two procedures.

**Conclusions:**

The present systematic review demonstrates that both LEEP and CKC are safe and effective for the conservative treatment of ACIS. LEEP appears to be as equally effective as CKC regarding the residual and recurrence rates. Due to the findings showing that LEEP achieves comparable oncologic outcomes with fewer obstetric complications to that of CKC, LEEP may be the preferred option in patients whose fertility preservation is important. However, further prospective studies with a larger sample size and longer follow-up periods are needed to establish the superiority of either procedure.

## Introduction

Cervical adenocarcinoma in situ (ACIS), first described by Hepler et al. in 1952 [1], is a precursor lesion for invasive cervical adenocarcinoma. In recent decades, a considerable reduction in squamous cervical cancer in more economically developed countries has occurred, concurrent with widespread cervical screening using cytology combined with human papillomavirus (HPV) testing. In contrast, the relative incidence of cervical adenocarcinoma has increased, now accounting for 25–30% of all invasive cervical cancers [2]. This increased prevalence is also found in ACIS, especially in younger women [3,4].

At present, hysterectomy remains the treatment of choice for women who have completed childbearing [5]. However, with more women delaying childbirth and the fact that the mean age of patients with ACIS is 37 years [4], many patients desire more conservative treatment. As the 2016 NHS Cervical Screening Programme confirmed [6], fertility-sparing treatment with conization is recommended for those wishing to retain fertility. Conservative management options for ACIS include loop electrosurgical excision procedure (LEEP) or large loop excision of the transformation zone (LLETZ), cold-knife conization (CKC), and straight wire excision of the transformation zone (SWETZ). SWETZ is applied less often [7,8], and currently the two main types for ACIS are LEEP and CKC. CKC has been the traditional procedure and is usually performed under general or regional anesthesia in a hospital setting with significantly higher costs. Compared to CKC, LEEP is usually performed under regional anesthesia in an outpatient low-cost clinic setting. Identification of the superior conservative procedure has become a hot topic in the treatment of ACIS.

Recent years have seen an increase in studies reporting effective conservative treatments of ACIS [2,9,10]. Some retrospective studies have compared treating ACIS conservatively with CKC and LEEP. However, these studies are inconsistent regarding the therapeutic efficacy associated with the two procedures. Previous studies and systematic reviews have favored CKC over LEEP for the treatment of ACIS [11–13], whereas recent studies showed that LEEP appears to be as equally effective as CKC [2,14], achieving the same rates of negative margins, diagnosis of invasive cancer, and recurrence of ACIS or invasive cancer. However, it has yet to be established whether LEEP is as effective as conventional CKC for the treatment of ACIS. The recently published American Society for Colposcopy and Cervical Pathology (ASCCP) guidelines do not make any recommendations about CKC or LEEP as the preferred therapy option, although the wording was changed from favoring CKC over loop excision in 2001 to allowing diagnostic excision using any modality including larger loops in 2006 [5]. For these reasons, a more comprehensive systematic review of original studies and a meta-analysis of positive margin and recurrence rates are urgently needed. The objective of this systematic review was to evaluate the efficacy and safety of LEEP versus CKC in the conservative surgical treatment of ACIS to guide management options for women who have not completed childbearing.

## Materials and Methods

### Search strategy

Systematic searches were performed using the MEDLINE, EMBASE, Cochrane and China National Knowledge Infrastructure (CNKI) databases to identify all articles published until December 2015 involving patients with ACIS treated with CKC or LEEP. The searches were restricted to English or Chinese literature and human studies. The text words used included “adenocarcinoma in situ of the cervix”, “glandular dysplasia of the cervix”, “large loop excision of the transformation zone (LLETZ)”, “loop electrosurgical excisional procedure (LEEP)”, and “cold knife conization”. Searches of the title and abstract of each publication were independently conducted by YMJ and LL to determine the potentially relevant studies.

### Study selection and data extraction

We included all studies that compared women who had been treated with LEEP to women who underwent CKC as the first treatment procedure. We excluded studies of case reports, unpublished works for lack of details, and studies with small sample sizes (n<10) to avoid selection bias. Additionally, we excluded studies with patients who were given a diagnosis only by cervical cytology of glandular dysplasia or hyperplasia or patients who had carcinoma (squamous or adenocarcinoma) concomitantly. The primary outcome included the rates of positive margins, residual disease, and recurrent disease. Positive margins, in relation to involvement with ACIS, were evaluated after the treatment of LEEP or CKC in the analysis. Recurrence or residual disease was assessed only with reference to ACIS or adenocarcinoma. Additional clinical outcomes that were extracted from the studies included the following: age, mean follow-up time, results of endocervical curettage (ECC), and pregnancy outcome. To determine the validity of the studies, the Newcastle-Ottawa Scale was used to assess the quality of the included studies[15]. The meta-analysis was performed in two groups: the experimental (LEEP or LLETZ) and control (CKC) groups. The data were independently extracted by YMJ and LL from each involved study, and any disagreements were resolved by consensus with a third review author (CXC) as necessary.

### Data synthesis

We extracted data from the experimental group and the control group for every observed outcome. The meta-analysis focused on the outcomes from two or more studies. Relative risks and 95% confidence intervals (CIs) were calculated with Revman 5.3 software (The Nordic Cochrane Centre, Copenhagen, Denmark), and heterogeneity was quantified using I^2^ statistics and P values [16]. Because there were no randomized controlled studies, a random effects model was used for the meta-analysis even if heterogeneity might be accepted (P>0.10, or I^2^<50%). Statistical significance was defined as a P-value less than 0.05. Funnel plot analysis to test for publication bias was performed if more than 10 studies were included in the review.

Because no identifiable and individual patient data were used in this meta-analysis, research ethics approval was not required.

## Results

### Study identification and selection

**Fig 1:**
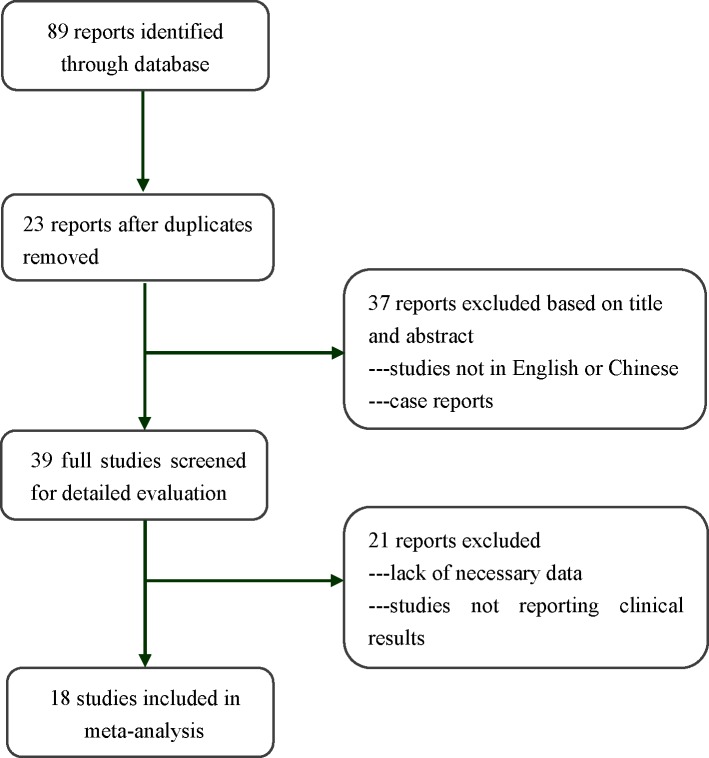
Study selection and exclusion process.

Of 89 potentially relevant eligible studies, 18 retrospective cohort studies that satisfied the inclusion criteria were included in this systematic review. Fig 1 shows the flow diagram for the literature search. Table 1 lists the main characteristics of the 18 included studies, representing 1,559 patients who underwent treatment. These 18 studies represented work conducted primarily in Europe and North America, as well as a single study from Asia [13]. No studies from South America or Africa were identified, and all of the studies were published in English. The sample sizes ranged from 33 to 338 (a total of 607 in the CKC group and 952 in the CKC group). The follow-up period ranged from 1 to 286 months. The mean age of the women included in the studies was generally less than 40 years, with only one study reporting a mean age of 45.1 years [13]. Table 2 shows the Newcastle-Ottawa scores for the risk of bias assessment of the included studies, and the 18 studies were rated with a total score of greater than 5, indicating a high risk of bias.

**Table 1:** Characteristics of the included studies.

Source	Country	Intervention		Histology	Study design	Study period	Follow-up period	Quality score*
		LEEP	CKC					
Munro 2015 [10]	Austria	107	231	ACIS	retrospective study	2001 to 2012	<1 year to 11.8 years	9
Latif 2015 [2]	USA	30	48	ACIS	retrospective study	1997 to 2011	2–168 months	9
Baalbergen 2015 [17]	Netherlands	45	65	ACIS	retrospective study	1989 to 2012	1–217 months	8
Taylor 2014 [18]	USA	15	37	ACIS	retrospective study	1998 to 2011	mean 32 months	7
Costales 2013 [12]	USA	62	110	ACIS	retrospective study	1983 to 2011	0.3–286.5 months	7
Hanegem 2012 [14]	USA	54	58	ACIS	retrospective	1998 to 2010	3–145 months	9
Kietpeerakool 2012 [13]	Thailand	34	20	ACIS	retrospective study	1998 to 2010	10–144 months	7
Costa 2012 [19]	Italy	60	74	ACIS	retrospective study	2004 to 2011	mean 40.9 months	9
DeSimone 2011 [20]	USA	17	24	ACIS	retrospective study	1990 to 2005	40 months	7
Bull-Phelps 2007 [21]	USA	32	69	ACIS	retrospective study	1993 to 2001	4–148 months	8
Hwang 2004 [22]	Canada	23	20	ACIS	retrospective study	1980 to 2002	1–248 months	6
Kennedy 2002 [23]	USA	30	27	ACIS	retrospective study	1994 to 2001	1–165 months	8
Soutter 2001 [24]	UK	43	10	ACIS	retrospective study	1986–2000	0–543 weeks	6
Kuohung 2000 [25]	USA	9	39	ACIS	retrospective study	1990 to 1999	NA	6
Azodi 1999 [26]	USA	8	25	ACIS	retrospective study	1988 to 1996	mean 38 months	9
Denehy 1997 [27]	USA	13	24	ACIS	retrospective study	1980 to 1996	1–72 months	6
Wolf 1996 [28]	USA	7	47	ACIS	retrospective study	1984 to 1993	17–132 months	9
Widrich 1996 [29]	USA	18	24	ACIS	retrospectivestudy	1980 to 1994	3–177 months	7

* Quality assessment based on the Newcastle-Ottawa Scale.

NA, not available.

**Table 2:** Assessment of study quality.

Source	Study design		Quality indicators from the Newcastle-Ottawa Scale									
			1	2	3	4	5	6	7	8	9	Score
Munro 2015 [10]	R	*		*	*	*	*	*	*	*	*	9
Latif 2015 [2]	R	*		*	*	*	*	*	*	*	*	9
Baalbergen2015 [17]	R	*		*	*	*	*		*	*	*	8
Taylor 2014 [18]	R	*		*	*	*			*	*	*	7
Costales 2013 [12]	R	*		*	*	*			*	*	*	7
Hanegem 2012 [14]	R	*		*	*	*	*	*	*	*	*	9
Kietpeerakool 2012 [13]	R	*		*	*	*			*	*	*	7
Costa 2012 [19]	R	*		*	*	*	*	*	*	*	*	9
DeSimone 2011 [20]	R	*		*	*	*			*	*	*	7
Bull-Phelps 2007 [21]	R	*		*	*	*	*		*	*	*	7
Hwang 2004 [22]	R	*		*	*				*	*	*	6
Kennedy 2002 [23]	R	*		*	*	*		*	*	*	*	8
Soutter 2001 [24]	R	*		*	*	*				*	*	6
Kuohung 2000 [25]	R	*		*	*				*	*	*	6
Azodi 1999 [26]	R	*		*	*	*	*	*	*	*	*	9
Denehy1997 [27]	R	*			*	*			*	*	*	6
Wolf 1996 [28]	R	*		*	*	*	*	*	*	*	*	9
Widrich 1996 [29]	R	*		*	*	*			*	*	*	7

Risk of bias was assessed with the Newcastle–Ottawa Scale. A score of 6 or more (out of 9) indicates a low risk of bias.

For cohort studies, 1 indicates exposed cohort truly representative; 2, non-exposed cohort drawn from the same community; 3, ascertainment of exposure; 4, outcome of interest; 5, cohorts comparable on basis of age; 6, cohorts comparable on other factor(s); 7, quality of outcome assessment; 8, follow-up long enough for outcomes to occur; and 9, complete accounting for cohorts.

R = Retrospective cohort.

### Positive margin rate

**Fig 2:**
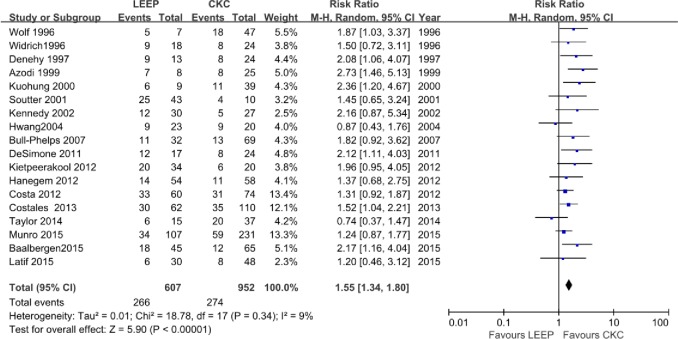
Comparison of LEEP and CKC in positive margin rate.

All 18 included studies reported the rate of positive margins, and the results of the individual studies varied. Ten [2,10,14,18–20,22–24,29] included studies did not report any significant differences for the treatment of ACIS, whereas the remaining 8 [12,13,17,21,25–28] described significantly higher positive margins rates for LEEP than for CKC. The prevalence of positive margins after LEEP was 266 among 607 women (44%); the prevalence after CKC was 274 among 952 women (29%). The pooled meta-analysis of the overall positive margins exhibited a significantly different outcome (RR, 1.55; 95% CI, 1.34–1.80, P<0.00001) without significant heterogeneity across the studies (P = 0.34) (Fig 2).

### Residual disease rate

**Fig 3:**
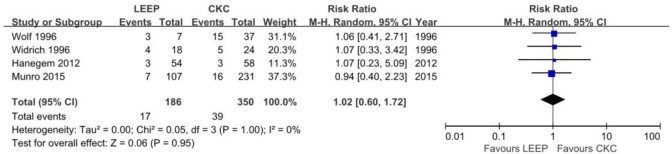
Comparison of LEEP and CKC in residual rate.

**Fig 4:**
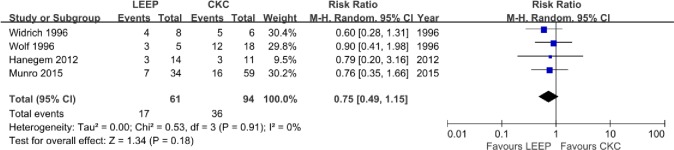
Comparison of LEEP and CKC in residual rate associated with positive margins.

Of the 18 studies, 4 [10,14,28,29] described the rate of residual disease for ACIS following CKC or LEEP. The results of the individual studies conflicted. Widrich et al. [29] showed that the residual rate after LEEP was significantly higher than that after CKC with a small sample size. However, there were no significant differences between LEEP and CKC in the proportion of residual ACIS reported by the other three studies. The prevalence of residual disease after LEEP was 17 among 186 women (9.1%); in contrast, the prevalence of residual disease was demonstrated in 39 of 350 (11%) patients after CKC in the re-cone or hysterectomy cases. The pooled meta-analysis of overall residual disease showed no significant differences (RR, 1.02; 95% CI, 0.60–1.72, P = 0.95) without significant heterogeneity (P = 1.00) (Fig 3). Residual disease among all the patients with positive margins was found in 17 of 61 (28%) women treated with LEEP and 36 of 94 (38%) women treated with CKC; however, the meta-analysis also exhibited no significant differences (RR, 0.75; 95% CI, 0.49–1.15, P = 0.18) without significant heterogeneity (P = 0.91) (Fig 4). For patients with negative margins who underwent the two procedures, the residual rate was lower than in patients with positive margins, although no significant difference was identified.

### Recurrence rate

**Fig 5:**
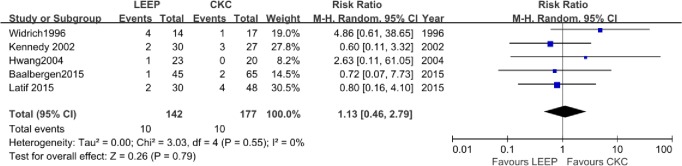
Comparison of LEEP and CKC in recurrence rate.

Of the 18 studies, 5 [2,17,22,23,29] reported recurrence rates with conservative therapy and did not describe any significant difference between CKC and LEEP. Recurrent ACIS following LEEP was reported in 10 of 142 (7.0%) cases compared to 10 of 177 (5.6%) cases following CKC. The pooled meta-analysis for the overall recurrence rates assessed in the five studies showed that there was no evidence of a significant difference following LEEP compared with CKC (RR, 1.13; 95% CI,0.46–2.79; P = 0.79) with no significant heterogeneity across the studies (P = 0.55, I^2^ = 0%) (Fig 5). Moreover, Munro et al. [10] reported that none of the included 39 patients, who received only surveillance without subsequent excision after undergoing positive margins, developed recurrent ACIS or cervical adenocarcinoma. Baalbergen et al. [17]found that recurrence after conservative therapy by CKC or LEEP was not significantly different than after radical therapy by hysterectomy with a mean follow up of 45–62 months (P = 0.56).

### Other outcomes

Of the 18 studies, 3 [17,21,29] studies mentioned results of pregnancy outcomes after the initial excision. Bull-Phelps et al. [21] reported that 35 of 101 women with ACIS had a total of 49 gestations during surveillance, and there were no differences in pregnancy outcome with regard to the type of initial cone biopsy. However, another two studies [17,29] were unable to acquire these results. In these studies, the overall pregnancy rate following conization varied from 28–47%.

Eight studies [12–14,20,23,26,27,29] reported the results of ECC performed concurrently with the initial excisional procedure. However, none of the 8 studies was able to detect a difference between ECC and the two excisions.

**Fig 6:**
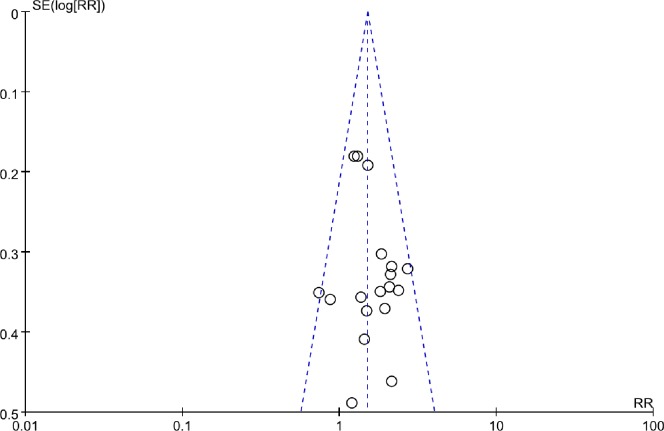
Funnel plots for publication bias for RR of positive margin rate.

The funnel plot assessment including all studies (positive margin rate) has revealed no evidence of possible publication bias (Fig 6).

## Discussion

With the rising incidence of ACIS in young women as the mean age in this meta-analysis was generally less than 40, there has been a tendency to treat with more conservative treatment, and the appropriate selection of conservative treatment type has come to the forefront of treatment literature. To our knowledge, this study is the first to use a meta-analysis of the previously reported studies to evaluate the employment of LEEP versus CKC in the conservative treatment of ACIS. In this review, we found 18 retrospective cohort studies in which LEEP was compared with CKC for ACIS. Differences between the included studies in terms of their setting, patient characteristics, and efficacy were observed. No significant heterogeneity was detected across studies for any of the evaluated data.

Controlling the positive margin rate of resection at a level as low as possible is difficult for the conservative treatment of ACIS. Three previous systematic reviews [4,11,30] favored CKC over LEEP in women with ACIS because LEEP resulted in a higher percentage of positive margins. The current analysis indicated that LEEP was associated with a 1.55-fold increase in the risk of positive margins compared with CKC for ACIS. A recent systematic review in 2014 by Baalbergen and Helmerhorst [11] found a clinically significantly higher rate (51%) of incomplete excision with LEEP than in CKC (30%). In our study, although the positive margins rate of 29% (274/952) after CKC was similar to that described previously and a significant difference was exhibited in the total studies between the two procedures, we found that the rate after LEEP was decreased to 44% (266/607), which seemed to show the tendency of preferred employment of LEEP for ACIS.

The residual and recurrence rates are perhaps the most important criteria for the conservative treatment of ACIS. As reported in other studies [14,28], patients with positive margins are more likely to have residual disease. Both Salani et al. [4] and Baalbergen and Helmerhorst [11] reported that nearly 50% of patients with positive margins were found to have residual ACIS on the repeated specimen, although no data are available about the differences between the treatment of CKC and LEEP. Our literature search, for the first time, found no significant differences in the residual rate associated with ACIS patients regardless of whether the margins were positive or negative after the two procedures. Moreover, the residual rates with conservative treatment in our review [17/61 (28%) with LEEP versus 36/94 (38%) with CKC, respectively] were much lower than previous reviews that reported an approximate 50% incidence in the residual after conization with positive margins. One explanation for this reduction in the residual rates is that conditions during the surgical procedures could have been better controlled. Therefore, ACIS patients who need to retain their fertility could reap the benefits of technological progress; however, a larger sample size of studies is necessary to further confirm these findings.

According to previous literature, the risk of recurrence is between 0% and 47% after conservative treatment. In our review, recurrent ACIS after LEEP was reported in 10 of 142 (7.0%) cases compared to 10 of 177 (5.6%) cases after CKC, comparable to the meta-analysis of Salani et al. [4] in 2009 that showed a 5% (34/671) recurrence rate after conservative therapy (CKC or LEEP). Our meta-analysis indicated that no significant differences were observed in the rates of recurrence with ACIS disease between the LEEP and CKC groups, which suggested that LEEP is as effective as CKC in surgical treatment. However, the subdivision of recurrence after negative and positive margins in the two treatments was not indicated as precise recurrence was reported only in one study. Moreover, the duration time of follow-up was different in the included studies, which may have caused a bias, and longer follow-up periods would be beneficial.

Eight included studies evaluated the performance of ECC concurrently with the initial excisional procedure, and none of the 8 studies was able to detect a difference between ECC and the two excisions. However, Costales et al. [12] found that 6/11 (60.0%) patients with a positive ECC showed residual ACIS in the specimen after subsequent conization. Similar results were reported by Tierney et al. [31] who showed that in cases where the ECC was positive for the presence of ACIS, 14 (78%) had residual ACIS, and 3 (17%) had invasive adenocarcinoma. These studies support that regardless of the treatment type, the addition of ECC for ACIS provides valuable prognostic information concerning the risk of residual disease.

Both treatments have been associated with preterm delivery, preterm premature rupture of membranes, and other adverse outcomes in subsequent pregnancies. In the included studies for ACIS, pregnancy outcome was not different with regard to the type of initial conization, and the most likely explanation for this result may be the limited sample. However, a large meta-analysis [32] showed that CKC was associated with an increased risk of preterm delivery (OR 2.8) compared to LEEP (OR 1.7). A large randomized, prospective trial [33] also found that the rate of preterm delivery among the LEEP group was 5%, whereas that for the CKC group was 11%. Thus, LEEP seems to be more beneficial for future pregnancies than CKC, although future studies are needed to identify which procedure can cure ACIS disease and also exert a less adverse pregnancy outcome. LEEP is by far the most popular procedure for cervical surgery for its clinical advantages. Given that CKC may increase the risk of adverse pregnancy outcomes compared to LEEP and that residual/recurrence rates of ACIS are comparable for both treatments, LEEP may be the preferred option in patients whose fertility preservation is important.

Several limitations of this systematic review should be considered in interpreting the presented data. First, of all the studies included in this review, the numbers of patients included in the older studies were much lower than those in the studies conducted more recently. Thus, it is difficult to conclude whether these trials reached therapeutic performance. Second, all studies included in the review were retrospective studies; indeed, prospective and randomized studies are difficult to conduct because of the low incidence of ACIS. Therefore, there might have been some confounders that were not recognized or controlled. In addition, patient age and follow-up time varied among the included studies, and these differences may have affected the results. Moreover, because no more details were obtained from the included studies, the outcomes between the two procedures cannot be stratified by age, especially in post-menopausal woman. Finally, it is possible that the exclusion of some missing and unpublished data might have caused a bias in the effect; the exclusion of non-English and non-Chinese language studies may also have led to publication bias.

In conclusion, the present systematic review demonstrates that both LEEP and CKC are safe and effective for the conservative treatment of ACIS. The risk of positive margins with LEEP tended to be comparable with that with CKC. Moreover, LEEP appears to be as equally effective as CKC regarding the residual and recurrence rates, which are perhaps the most important features considered for conservative treatment. Due to the evidence showing that LEEP achieves comparable oncologic outcomes to those of CKC, with fewer obstetric complications, LEEP may be the preferred option in patients whose fertility preservation is important. However, further prospective studies with a larger sample size and longer follow-up period are needed to establish the superior procedure.

## Supporting Information

S1 Checklist A PRISMA checklist for this systematic review. (DOC)
